# EVALUATION OF OUTCOMES IN INTERVENTION RANDOMIZED CLINICAL TRIALS - DISTAL RADIUS FRACTURES

**DOI:** 10.1590/1413-785220233103e267872

**Published:** 2023-09-08

**Authors:** Davi Amorim Meira, Lukas Eiki Moriyama, Cássio Conceição Santana Santos, Fernando Delmonte Moreira, Alex Guedes, Enilton de Santana Ribeiro de Mattos

**Affiliations:** 1 Escola Bahiana de Medicina e Saúde Pública, Salvador, BA, Brazil.; 2 Fundação Universidade Federal da Grande Dourados, Faculdade de Medicina, Dourados, MS, Brazil.; 3 Universidade Federal da Bahia, Faculdade de Medicina da Bahia, Salvador, BA, Brasil.; 4 Universidade Federal da Bahia, Complexo Hospitalar Universitário Professor Edgard Santos, Programa de Residência Médica em Ortopedia e Traumatologia, Empresa Brasileira de Serviços Hospitalares, Salvador, BA, Brazil.; 5 Universidade Federal da Bahia, Unidade do Sistema Neuro-Músculo-Esquelético, Empresa Brasileira de Serviços Hospitalares, Salvador, BA, Brazil.; 6 Universidade Federal da Bahia, Faculdade de Medicina da Bahia, Departamento de Cirurgia Experimental e Especialidades Cirúrgicas, Salvador, BA, Brazil.

**Keywords:** Outcome Assessment, Health Care, Radius Fractures, Randomized Controlled Trials as Topic, Wrist, Avaliação de Resultados em Cuidados de Saúde, Fraturas do Rádio, Ensaios Clínicos Controlados Aleatórios como Assunto, Punho

## Abstract

**Objectives:**

Describe the frequency and types of outcomes in randomized clinical trials (RCT) of intervention for distal radius fractures, analyze how confusing outcome presentations can lead to misinterpretations, and suggest strategies to improve the reader's understanding of the decision-making process.

**Methods:**

A retrospective study was conducted through a systematized search on the PubMed® database in the last 10 years, in which only intervention RCT was included for distal radius fractures, and outcomes were analyzed.

**Results:**

Of the primary outcomes analyzed in the 75 selected articles, 46.6% were classified as clinical outcomes, 20% as surrogate, 30.6% as composite, 1.3% as complex scales, and 1.3% as safety outcomes. 34.7% of the articles did not report adverse events.

**Conclusion:**

The presentation of outcomes with little clinical relevance represented more than half of the sample (53.4%) - such studies can harm the reader since they confuse the interpretation of scientific evidence; the Core Outcome Measures in Effectiveness Trials (COMET) initiative could help health professionals in understanding and selecting the most appropriate therapeutic interventions for patients. ***Level of Evidence III; Retrospective comparative study*** .

## RESUMO

**Objetivos:**

Descrever a frequência e os tipos de desfechos em ensaios clínicos randomizados (RCT) de intervenção para fraturas distais do rádio, analisar como apresentações confusas de desfechos podem levar a interpretações equivocadas e sugerir estratégias para melhorar a compreensão do leitor sobre o processo de tomada de decisão.

**Métodos:**

Foi realizado estudo retrospectivo mediante busca sistematizada na base de dados PubMed® nos últimos 10 anos, na qual foram incluídos apenas RCT de intervenção para fraturas do segmento distal do rádio, cujos desfechos foram analisados.

**Resultados:**

Dos desfechos primários analisados nos 75 artigos selecionados, 46,6% foram classificados como desfechos clínicos, 20% como substitutos, 30,6% como compostos, 1,3% como escalas complexas e em 1,3% como desfechos de segurança. 34,7% dos artigos não reportaram eventos adversos.

**Conclusão:**

A apresentação de desfechos com pouca relevância clínica representou mais da metade da amostra (53,4%) - tais estudos podem prejudicar o leitor, uma vez que confundem a interpretação das evidências científicas; a iniciativa Core Outcome Measures in Effectiveness Trials (COMET) auxilia os profissionais de saúde na compreensão e seleção das intervenções terapêuticas mais adequadas para os pacientes. ***Nível de Evidência III; Estudo retrospectivo comparativo*** .

## INTRODUCTION

The distal radius is the most common fracture site in the upper limbs.^1^ The mechanisms of injury range from falls of one's own height to high-energy traumas.^2 – 4^ The distribution of distal radius fractures is bimodal, accompanying the gender and age of the patient's, being more frequent in young adult men (associated with high-energy trauma), and in elderly women due to falls from their own height (osteoporosis-related). Shauver et al.^5^ estimated that the cost of hospitalizations for these fractures in the elderly to the U.S. public health system was $170 million in 2007.

Diverse intervention randomized clinical trials (RCT) have been conducted, aiming to achieve better alternatives for the treatment of distal radius fractures. Viergever et al.^6^ observed that there has been a substantial increase in the number of RCT, not necessarily accompanied by an increase on quality, underestimating the potential benefits that these studies can promote. It is known that RCT, although located at the top of the evidence pyramid and important in decision-making process, have high associated costs and demands great efforts on the part of research teams.^7 , 8^ Thus, to mitigate expenses and simplify the work, many researchers choose to use few clear outcomes that do not translate into clinical improvement for patients.^9^

Outcomes can be defined as measures of the effects of an intervention. Smith et al.,^10^ analyzing the results of an online Delphi survey of 48 UK Clinical Research Collaboration registered Clinical Trials Units, concluded that research into methods to boost recruitment in trials, methods to minimize attrition, and methods for choosing appropriate outcomes to measure are priority topics for methodological research. In this context, we can observe a correlation with the study of Heneghan et al.,^9^ that highlights the need to select clinical outcomes in RCT, to promote papers that are capable of translating improvements in patients’ health status.

The objectives of this paper are to describe the frequency and types of outcomes in randomized clinical trials (RCT) of intervention for distal radius fractures, to analyze how confusing outcome presentations can lead to misinterpretations, and to suggest strategies to improve the reader's understanding of decision-making.

## METHODS

A retrospective study was conducted through a systematized search on the PubMed® database in the last 10 years, being included only intervention RCT for distal radius fractures which outcomes were analyzed. A search was carried out in the PubMed® database using the strategy described in the Table 1 , without language restriction. Papers that did not constitute intervention RCT, duplicate papers or which that addressed anatomical sites other than distal radius were excluded. Two independent authors selected the articles by title and abstract using the Rayyan© web applicative according to the inclusion criteria, and possible divergences were resolved by consensus. The selected articles were read in full, and the primary outcomes classified according to the criteria proposed by Heneghan et al.^9^

**Table 1:** Search strategy.

N	Search Strategy
#1	Radius Fracture [Title/Abstract] OR Fracture, Radius [Title/Abstract] OR Fractures, Radius [Title/Abstract]
#2	Randomized Controlled Trial [Publication Type]
#3	#1 and #2

The search was carried out in PubMed® database on 09/01/2022, and a total of 120 papers were found. After applying the exclusion criteria, 75 articles remained.

## RESULTS

**Figure 1:**
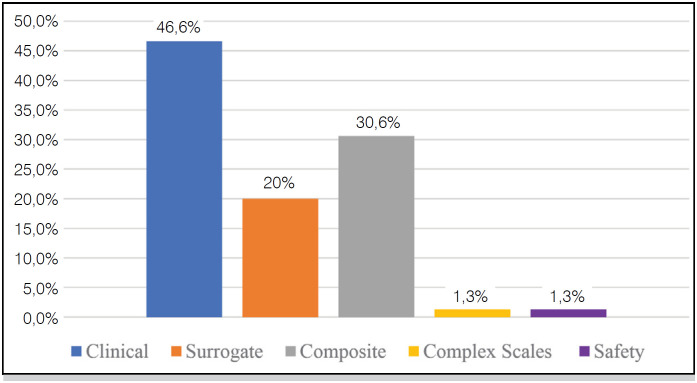
Number of Papers X Primary Outcome Types.

**Figure 2:**
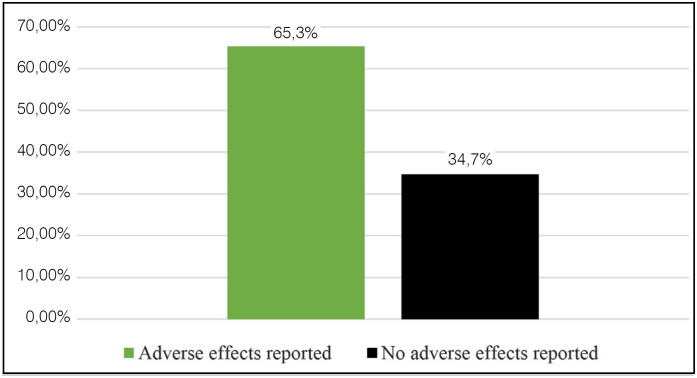
Reporting of adverse effects in the studied sample.

Among the 75 selected RCT, we found 35 articles with clinical outcomes (46.6%), 15 articles with surrogate outcomes (20%), 23 articles with composite outcomes (30.6%), 1 article (1.3%) with complex scales and 1 article (1.3%) with safety outcome ( Figure 1 ).49 articles (65.3%) reported adverse events, and 26 articles (34.7%) did not ( Figure 2 ).

## DISCUSSION

The purpose of much of the scientific production in the health area is the validation of practices that promote advances in patient care, thus ensuring an improvement in quality of life.^9 , 11 , 12^ From this perspective, RCT have the function of testing hypotheses and evaluating them based on chosen outcomes according to the purpose of the study. A choice of outcomes requires a lot of attention, constituting an essential part of the study planning, since according to the objective of the study, whether to analyze the pre-test probability or test the effectiveness of a conduct, certain parameters will be more adequate than others.^10^

Heneghan et al.^9^ explicit that one of the reasons why RCT cannot translate benefits for patients is precisely the mistaken choice of outcomes, opting for unclear ones, without relevance in clinical practice. The authors classify the outcomes into clinical, surrogate, composite and subjective, besides mentioning the use of complex scales in the evaluation of interventions. Clinical outcomes are those capable of reflecting real-world configurations and the patients’ true needs, being therefore related to quality of life after intervention, of greater relevance in medical practice. Surrogate outcomes are used to infer or predict outcomes of clinical relevance, being useful in the evaluation of pre-test probability in phase 2 studies but should not be used to define medical conducts. Composite outcomes are characterized by the evaluation of factors combined in the measure of the outcome, promoting a reduction in the sample sizes, besides presenting potential for confusing interpretation of the results due to the combination of factors. Subjective outcomes are marked by the need for judgment by the researcher or are reported by patients. The use of complex scales is related to the combination of signs and symptoms in scales created by the authors of the study, which becomes problematic because these are not validated and reliable measurements as the RCT requires.

In our sample, we found that most articles (53.4%) used, in their primary outcomes, measures unable to translate the improvement in patients’ health, and, therefore, did not present greater importance in clinical practice. In total, 15 studies (20%) used surrogate outcomes, 23 studies (30.6%) used composite outcomes, one study (1.3%) used safety outcomes and one study (1.3%) used complex scales.

This wide range of articles using outcomes that do not adequately assess the patients’ clinical condition indicates that most intervention RCT that approaches distal radius fractures are not able to correctly translate an improvement in the patients’ health status. However, it cannot be affirmed that these studies are of no scientific importance, since the use of surrogate or composite outcomes may be unique in the early stages of randomized clinical trials, to estimate the pre-test probability, giving the researcher the ability to decide whether to continue with the research, since these outcomes require a shorter follow-up time than clinical outcomes.^12–14^

The surrogate outcomes are indirect measures used in order to estimate a clinical importance, and present as the main quality the fact that they are defined by means of continuous variables, easy to measure and of short-term response, which decreases the follow-up time of the studies.^13 , 14^ To determine the quality of a surrogate outcome, it should present a causal relationship between the intervention and the surrogate outcome and between it and the clinical outcome - this relationship should be the main route of action of the intervention on the clinical outcome.^13 , 14^ In the studied RCT, we can affirm that the surrogate outcomes were well chosen, since, for the most part, measures of joint amplitude and hand strength were used, directly related to limb functionality.

Rupp et al.^15^ evidenced that although surrogate outcomes were sufficient for FDA approval of new anti-cancer drugs, these medications were not able to increase patients’ survival or improve their quality of life; therefore, caution should be exercised in interpreting such outcomes.

The importance of composite outcomes lies in the decrease in the sample size needed to make statements, thus increasing the statistical power of work.^12 , 16^ Meanwhile, its impairment lies in the confusing interpretation of the results, since we cannot clearly state whether the intervention is effective.^9 , 16 , 17^ Thus, similarly to surrogate outcomes, compound outcomes contribute to simplify the work, increasing the speed of completion of the study, being useful to formulate hypotheses about the intervention.^16^ In the evaluated studies, compound outcomes, in most cases, combined measures of surrogate outcomes, such as range of motion and hand strength, with measures of clinical outcomes, such as limb functionality questionnaires.

The sample also presented two other studies, one evaluating safety outcomes and the other using complex scales in the analysis of outcomes. Safety outcomes are useful in early stages of RCT, when one wants to test whether the intervention can bring harm to the patient's health, being used in small samples composed of healthy individuals, seeking for frequent and serious events, besides being used also in the final phase, in order to make an analysis of the net benefit of the intervention.^18^ Moreover, complex scales are used in situations where there are no validated questionnaires to evaluate patients; are related to a great risk of bias, since they are created by the evaluators themselves, tending to a greater positivity of the paper.^9^

As previously mentioned, clinical outcomes are those capable of translating a real improvement in the patient's health status, being clinically relevant per se and, thus, RCT that use it are more appropriate to guide medical practice.^9 , 11 , 12^ In the studied sample, 46.6% (35) of the articles used clinical outcomes, mainly using parameters of limb functionality and quality of life. To access them, validated questionnaires such as DASH, QuickDASH, PRWE, MHQ, SF-36, in addition to analogue pain scale were used. However, the counterpoint of these methods is that they are considered subjective clinical outcomes, since they require the patient's response, appealing to individual subjectivity.^9^ Thus, it is a great challenge to evaluate patients clinically and objectively, since the main objective of the interventions is to restore functionality and promote increased quality of life, variables that are difficult to be objectively measured.

Regarding the report of adverse events in the studied sample, we observed that, of the 75 RCT analyzed, 26 did not do it, a number greater than one third of the papers in appreciation. It is essential that the complications resulting from a certain intervention are reported in the RCT, since this information is of great importance in clinical practice, allowing the reader to analyze its benefit-harm ratio.

A solution to the described problems is the Core Outcome Measures in Effectiveness Trials (COMET)^19^ - this initiative aims to facilitate the development and application of outcomes that should be measured and reported in clinical trials of a specific disease or experimental population. Its main role is the development of a guideline on how to select outcome measurement instruments for results included in a study. The proposal is of great importance because it recommends outcome measures that represent clinical efficacy, helping the researcher to choose the most appropriate therapeutic interventions. This initiative seeks to standardize such outcomes, facilitating the reader's understanding, as well as the realization of reviews and data joint analysis in a meta-analysis.

## CONCLUSIONS

Scientific papers which generate not clear outcomes to readers or have low clinical impact for patients represent an important problem described in the medical literature.

In the studied sample, which included the primary outcomes in 75 intervention RCT for distal radius fractures, 46.6% were considered as clinical outcomes, 20% as surrogate, 30.6% as composite, 1.3% as complex scales and 1.3% as safety outcomes. 34.7% of the articles did not report adverse events. The presentation of outcomes with little clinical relevance represented more than half of the sample (53.4%) - such studies can harm the reader since they confuse the interpretation of scientific evidence and the decision-making process on the part of health professionals, leading them to opt for interventions that do not bring real benefits to patients.

Measures such as those of COMET initiative for the selection of research outcomes could help health professionals in understanding and selecting the most appropriate therapeutic interventions for patients.
